# Pathway information on methylation analysis using deep neural network (PROMINENT): An interpretable deep learning method with pathway prior for phenotype prediction using gene-level DNA methylation

**DOI:** 10.1016/j.artmed.2025.103236

**Published:** 2025-08-29

**Authors:** Soyeon Kim, Laizhi Zhang, Yidi Qin, Rebecca I. Caldino Bohn, Hyun Jung Park

**Affiliations:** a Division of Pulmonary Medicine, Department of Pediatrics, School of Medicine, University of Pittsburgh, Pittsburgh, PA, United States; b Department of Human Genetics, School of Public Health, University of Pittsburgh, Pittsburgh, PA, United States

**Keywords:** Deep learning, DNA methylation, Phenotype prediction, Pathway, Gene

## Abstract

**Background:**

DNA methylation is a key epigenetic marker that influences gene expression and phenotype regulation, and is affected by both genetic and environmental factors. Traditional linear regression methods such as elastic nets have been employed to assess the cumulative effects of multiple DNA methylation markers on phenotypes. However, these methods often fail to capture the complex nonlinear nature of the data. Recent deep learning approaches, such as MethylNet, have improved the prediction accuracy but lack interpretability and efficiency.

**Findings:**

To address these limitations, we introduced Pathway Information on Methylation Analysis using a Deep Neural Network (PROMINENT), a novel interpretable deep learning method that integrates gene-level DNA methylation data with biological pathway information for phenotype prediction. PROMINENT enhances interpretability and prediction accuracy by incorporating gene- and pathway-level priors from databases such as Gene Ontology (GO) and Kyoto Encyclopedia of Genes and Genomes (KEGG). It employs SHapley Additive exPlanations (SHAP) to prioritize significant genes and pathways. Evaluated across various datasets, childhood asthma, idiopathic pulmonary fibrosis (IPF), and first-episode psychosis (FEP)—PROMINENT consistently outperformed existing methods in terms of prediction accuracy and computational efficiency. PROMINENT also identified crucial genes and pathways involved in disease mechanisms.

**Conclusions:**

PROMINENT represents a significant advancement in leveraging DNA methylation data for phenotype prediction, offering both high accuracy and interpretability within reasonable computational time. This method holds promise for elucidating the epigenetic underpinnings of complex diseases and enhancing the utility of DNA methylation data in biomedical research.

## 1. Background

DNA methylation is an epigenetic marker that is important for the normal development of mammals. Both genetic variation [1] and the environment [2] can alter DNA methylation, which plays a critical role in regulating gene expression. Thus, identifying the association between DNA methylation (DNAm) and phenotypes through epigenome-wide association studies (EWAS) is a pivotal step in understanding the complex interplay between genetics, the environment, and phenotypes [3]. In addition, Methylation Risk Scores (MRS) or methylation profile scores (MPS) using linear regression methods, such as the elastic net, have gained prominence for assessing the collective effect of multiple DNAm markers on phenotypes and, therefore, assessing individual risk for a disease such as type 2 diabetes or lung function [4]. The collective effects of DNA methylation on gene regulation and phenotypic outcomes are highly nonlinear because of the complex interactions among methylation sites and their context-specific roles in regulating gene expression. These interactions often involve intricate dependencies between methylation marks across different genomic regions as well as interactions with transcription factors and chromatin states. Traditional linear models are insufficient to capture these complexities because they assume additive or straightforward relationships that oversimplify the underlying biological mechanisms [5].

On the other hand, deep learning methods are uniquely suited to model such nonlinear patterns because of their ability to learn hierarchical representations of data and capture intricate dependencies at multiple levels. By leveraging architectures, such as neural networks, deep learning can uncover hidden patterns and relationships in high-dimensional methylation data, providing a more accurate and biologically meaningful representation of methylation effects. Thus, deep learning is an essential tool for understanding the collective impact of methylation on complex biological processes and diseases. A deep neural network (DNN) method, MethylNet, was proposed to accurately predict various phenotypes using DNAm by addressing the nonlinear nature of the data. By leveraging multilayered nonlinear activators in the model, MethylNet outperformed existing linear regression-based methods for predicting age, cancer subtype, and cell type proportions [6]. MethylNet also incorporates the SHapley Additive exPlanations (SHAP) explainer [7] to prioritize important CpG sites. Gomes et al. [8] introduced a deep learning approach aimed at reducing feature dimensionality through the utilization of statistical methods such as ANOVA and Random Forest before the deployment of deep learning models. This method demonstrated fast computational time and high accuracy on TCGA breast cancer dataset. However, the methodology has some limitations. Feature selection was performed prior to the division of data into training and testing sets, potentially leading to over-estimated prediction accuracies. In addition, the reported computation times did not account for the duration of the feature selection process, thus underestimating the total computation time.

Although the utilization of deep neural network (DNN) approaches has notably enhanced prediction accuracy, they still show three shared key limitations. First, the deep neural network (DNN) approach treats each CpG site as an independent feature, thereby neglecting explicit modeling in relation to genes. The gene-level methylation models over CpG level methylation have three advantages: prediction accuracy, interpretability, and computing time. i) Previous studies using gene-level methylation have shown that various machine learning algorithms, such as support vector machines and random forests, perform better when using gene-level methylation compared to CpG-level methylation in predicting various phenotypes such as rheumatoid arthritis and cancers [9,10]. In addition, a differentially methylated gene region approach identified unique CpGs that were not detected through CpG-wise methods [11], suggesting gene-based tests can increase the statistical power. This suggests that in the prediction problem, gene-level methylation can increase the prediction accuracy of the phenotype/diseases. ii) The gene-level methylation process allows us to interpret DNAm markers for gene-level downstream analyses. Previous methods typically select DNA methylation features at the CpG site level, which hinders biological interpretability and necessitates additional post hoc mapping of CpG sites to their associated genes. iii) Given the numerous DNAm markers in array data (e.g., 450 K, 850 K, or 950 K), optimizing parameters for the vast number of CpGs in the DNN is time-consuming. While Gomes et al. [8] incorporated feature selection before utilizing a DNN to reduce computation time, it would require a tremendous amount of computational time for feature selection if it was performed only in the training dataset, especially in a cross-validation setting. However, gene-level processing reduces the parameters to optimize the number of genes, thus significantly streamlining the computational effort required for training models. Second, existing DNN methods that utilize DNAm data typically overlook the incorporation of biological pathways into their models. As biological pathways represent sets of genes that interact with specific biological functions, comprehensive integration of pathway information could enhance both the interpretability and accuracy of phenotype predictions. This integration enables the DNN model to reflect complex molecular interactions that are crucial for understanding disease mechanisms. While this integration has been done in methods utilizing transcriptome data (STable 1), it has not been done using DNA methylation data because it is not intuitive to assign each CpG to gene pathways. Third, the current methodology using DNAm does not explicitly identify genes and pathways that influence phenotypes through DNA methylation. Previous methods such as MethylNet [6] or Gomes et al. [8] have identified CpG sites within the model, not genes or pathways. However, identifying key genes and pathways that influence disease prediction are crucial to understand biological mechanisms and to identify prognostic markers for each disease. Addressing these limitations would not only enhance the interpretability and accuracy of DNAm-based predictions, but also provide valuable insights into disease mechanisms.

**Fig. 1.**
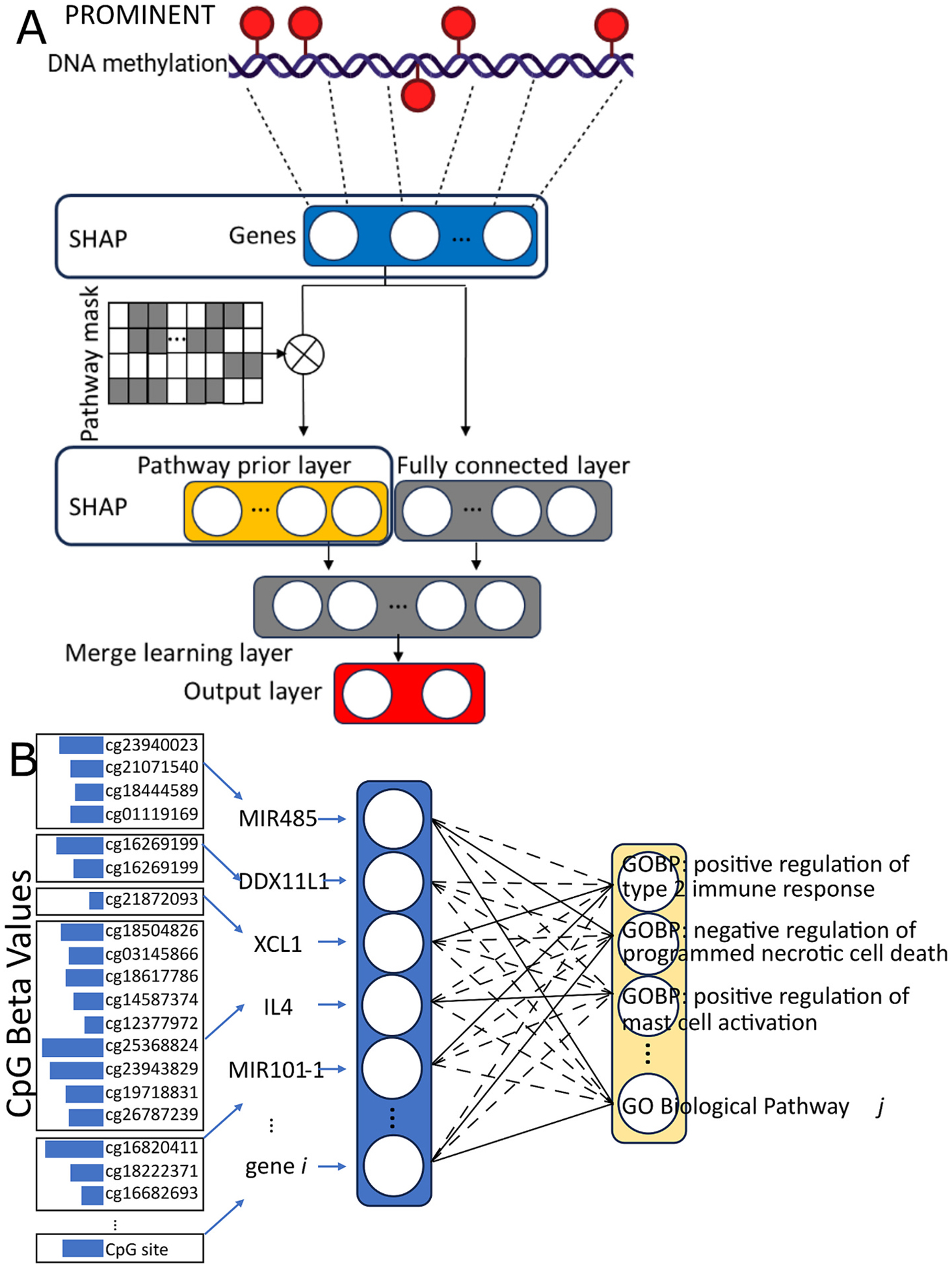
Overview of PROMINENT. (A) PROMINENT takes the input matrix of CpG methylation beta values, aggregates them into gene-level beta values, and trains in two layers, one with pathway prior and the other in the fully connected layer. Then, the divergent training process gets merged in the subsequent layer (merged learning layer) to produce the output layer value. (B) An example demonstrating how information is passed in PROMINENT, from CpG methylation levels to gene levels (blue arrow), to GO pathway prior. Connections are made from the gene-level values to GO pathways only when the genes are involved in a GO pathway (represented as a connected line).

To overcome these challenges, we introduced Pathway Information on Methylation Analysis using a Deep Neural Network (PROMINENT). To address the first limitation and consider gene-level methylation, we streamlined our approach by aggregating methylation information to the gene level. This enhances biological interpretability by allowing direct mapping of methylation signals to genes and facilitating downstream pathway enrichment analyses and functional interpretation. Also, the aggregation enables us to reduce computing time. To address the second limitation and incorporate prior biological knowledge, PROMINENT integrates biological pathway information from databases such as Gene Ontology (GO) and Kyoto Encyclopedia of Genes and Genomes (KEGG) by adding information on the additional computational layer. This integration is achieved through additional layers that consider pathway priors (Fig. 1A, B and SFig. 1). This structured representation enables PROMINENT to learn with biological context, improving the relevance and robustness of predictions. To address the third limitation and select important genes and pathways, PROMINENT incorporates both pathway layers and gene-level fully connected layers and then prioritizes genes and pathways by leveraging the SHAP explainer on the gene-level input layer and pathway-level layer. SHAP has been successfully used to estimate the contribution of input features in diverse DNN models. By placing SHAP explainers in both gene and pathway layers, PROMINENT enables the interpretation of methylation effects at both levels. This dual-layer attribution allows researchers to identify both key genes and their upstream pathways that contribute to phenotype prediction.

We evaluated the performance of PROMINENT across various pathologies and tissues by predicting the childhood asthma phenotype using DNAm data in peripheral blood mononuclear cells (PBMC), idiopathic pulmonary fibrosis (IPF) using DNAm data in lung tissue, and first-episode psychosis (FEP) using DNAm data in blood, in comparison with MethylNet and the elastic net approach, which is often used to calculate PRS/MPS. To investigate the effect of pathway prior consideration, we presented PROMINENT with two modules for comparison: one with pathway priors and one without.

In our study, we compared PROMINENT with MethylNet and ElasticNet because these methods are specifically tailored for DNA methylation data analysis and are widely recognized in the field. MethylNet is a neural network-based method that has shown high prediction accuracy in complex methylation data, whereas ElasticNet is a popular regression method with a regularization technique that offers a simpler structure and fast computational time. We did not compare PROMINENT with Gomes et al. [8] because it shares common limitations with MethylNet, and conducting feature selection across multiple training datasets using cross-validation is time-consuming.

In extensive comparisons, while PROMINENT demonstrated superior performance compared to MethylNet and better or similar performance compared to the elastic net in terms of overall prediction accuracy, PROMINENT adeptly prioritizes genes and pathways that are pivotal to disease pathogenesis, a capability that is absent in other methodologies. Moreover, it surpasses MethylNet in terms of computational efficiency and achieves a computational time similar to that of elastic net. By explicitly integrating gene-level DNAm with pathway priors into DNN, along with SHAP, PROMINENT presents a promising approach for phenotype prediction and the elucidation of crucial genes and pathways implicated in disease etiology, all accomplished within a reasonable computational time.

## 2. Findings

### 2.1. Overview of PROMINENT

To predict disease status and prioritize genes and pathways using DNA methylation data, we integrate gene-level DNA methylation information and pathway knowledge into a deep neural network (DNN) model. First, we aggregate DNA methylation beta values from multiple CpG sites into gene-level representations (referred to as the preprocessing step). PROMINENT then feeds these gene-level values into the input layer, subsequently passing through the pathway prior layer, fully connected layer, merged learning layer, and classification output layer (Fig. 1A, SFig. 1). While the other layers adhere to a typical DNN architecture, the pathway layer uniquely consolidates methylation values at the gene level into pathway-level representations, thereby enabling biological interpretation and high prediction accuracy. For pathway information, we employed the Gene Ontology Biological Process (GOBP), the Gene Ontology Cellular Component (GOCC), REACTOME, and Kyoto Encyclopedia of Genes and Genomes (KEGG) databases (SFig. 2), respectively, whereas we also tested PROMINENT without the pathway prior layer for comparison (Fig. 1B). Unlike conventional DNN models lacking transparency, PROMINENT incorporates SHAP explainers in both the gene input and pathway layers to prioritize important genes and pathways that are relevant to the disease of interest. Using the SHAP explainer in the pathway layer, PROMINENT prioritized pathways directly without enrichment analysis. Furthermore, by merging the pathway layer with the gene-level fully connected layer, PROMINENT predicts phenotypes that rely not only on pathway knowledge, but also on gene-level information.

### 2.2. PROMINENT outperforms existing methods in predicting asthma using the gene-level methylation data

**Fig. 2.**
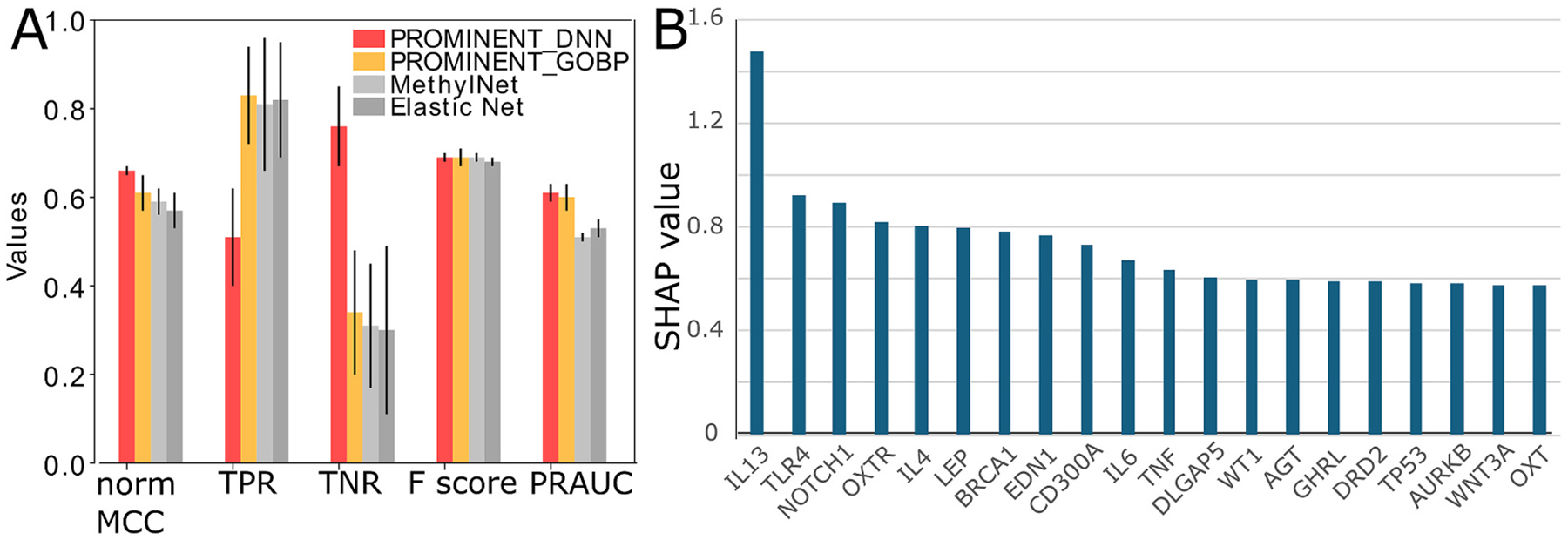
Performance Evaluation and Feature Importance in Predicting Asthma Using PROMINENT and Machine Learning Models. (A) Barplots showing different accuracy metrics to predict asthma vs. normal using PROMINENT_DNN (red), PROMINENT_GOBP (orange), MethylNet (light gray), and Elastic Net (dark gray) on the average of the nested 5-fold CV. Those measures are Normalized Matthew’ correlation coefficient (MCC), Norm MCC = (MCC + 1)/2, true positive rate (TPR), true negative rate (TNR), F1 score, and area under the precision-recall curve (PRAUC). Error bars are standard deviation bars based on the nested 5-fold CV experiments. (B) Barplot of SHAP-based importance of gene-level methylation for the asthma prediction (the sum of SHAP value across individuals for each gene).

To compare two modules of PROMINENT (with and without pathway information) with MethylNet and elastic net, we analyzed DNAm data from peripheral blood mononuclear cells (PBMC) of children with atopy and persistent asthma (cases, n=97) and without atopy or asthma (healthy control subjects, n = 97) (GSE40576) [12]. After QC and filtering, we obtained integrated CpG-level beta values within 20,493 gene-level beta values (see the Methods section). Because PROMINENT can control the weights between the pathway layer and the fully connected layer, we ran PROMINENT with GO pathways (PROMINENT_GOBP) or without the pathway-level prior (PROMINENT_DNN). We compared the four methods using the gene-level methylation data. In the nested 5-fold cross-validation, both versions of PROMINENT outperformed the other methods in terms of the Precision-Recall Area Under the Curve (PRAUC), normalized Matthews Correlation Coefficient (norm MCC), and True Negative Rate (TNR) (Fig. 2A). We deemed PRAUC and MCC important because they evaluate the performance of classification models, particularly for imbalanced datasets, and MCC is particularly reliable for binary prediction because it considers all aspects of identification performance, namely true positives, true negatives, false positives, and false negatives [13]. In addition, because TNR estimates how well the model identifies actual negative cases and avoids classifying them as positive (false positives), PROMINENT’s superior performance in TNR is beneficial for guiding and facilitating further experimental validation as a potential prognostic marker for childhood asthma.

In addition to its noteworthy performance in prediction accuracy, PROMINENT provides pathogenetic insights into possible methylation-mediated gene regulatory mechanisms in childhood asthma. For example, PROMINENT identified *IL13* and *IL4* with 1st and 5th SHAP contribution values (Fig. 2B, STable 2). *IL13* is hypomethylated in childhood asthma, and *IL4* expression is significantly associated with DNA methylation [12]. Also, *TLR4* (Toll-like receptor 4), which has the second highest SHAP value, is a key regulator of asthma exacerbation [14] that often leads to hospitalization or emergency room visits [15]. In addition, *NOTCH1*, which has the third highest SHAP value, maintains homeostasis in T cell cytokine production between Th1 and Th2 profiles during asthma. Furthermore, a gene identified using PROMINENT was found in a larger cohort, thereby demonstrating the sensitivity of PROMINENT. *LEP* was identified as the 6th strongest gene by PROMINENT in the analyzed dataset (n=194), and a previous larger study (the Isle of Wight, n=1313) found associations between methylation in the LEP gene and asthma as well as lung function [16].

### 2.3. PROMINENT enables downstream analyses originally not designed for methylation data

**Fig. 3.**
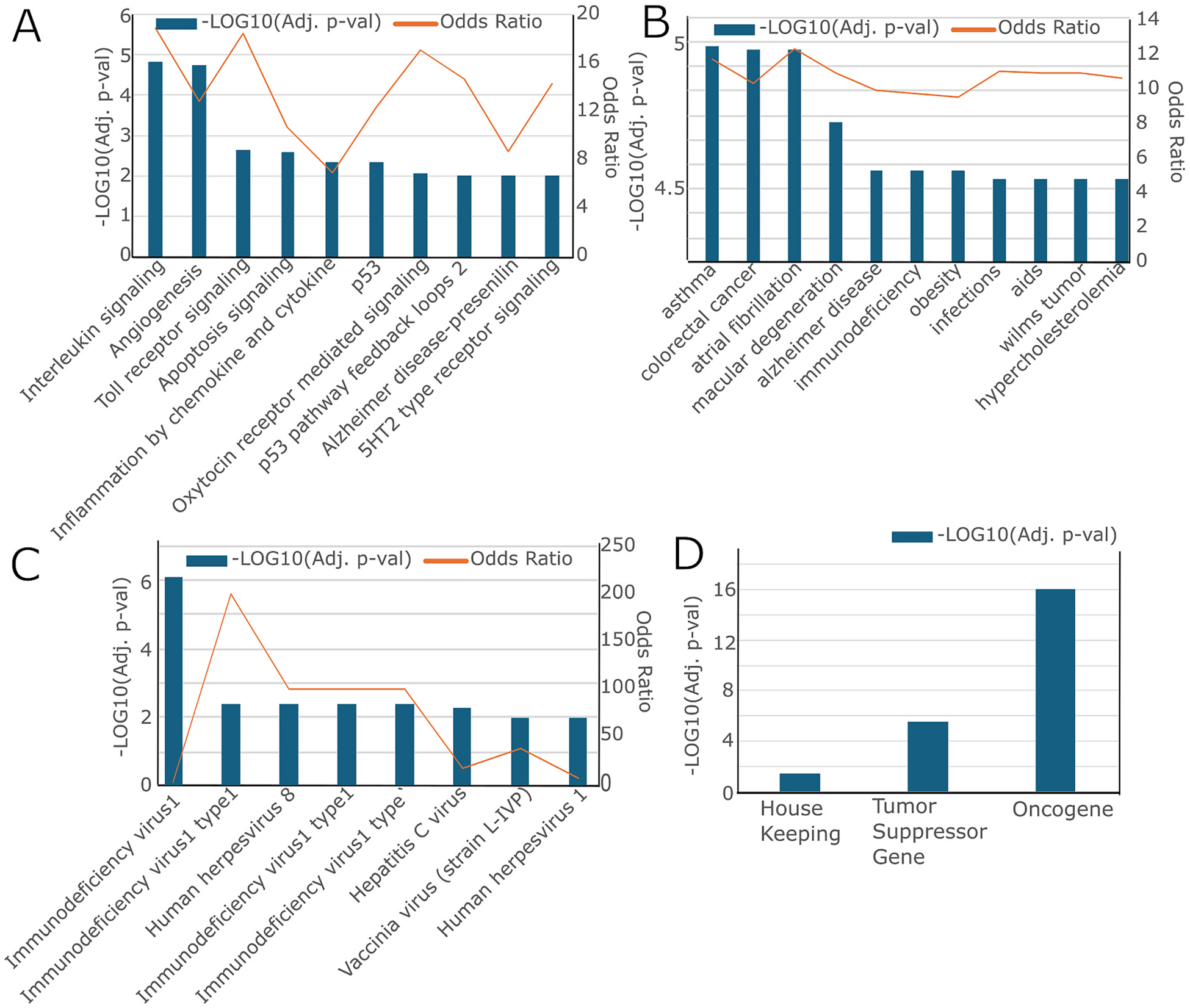
Downstream Gene-based Analysis on Asthma Using 200 Important Genes Prioritized by PROMINENT. (A) Barplot showing the enrichment of those genes in Panther classification database. Bar height indicates statistical significance (−log10 of adjusted *p*-value) and orange line indicates odds ratio calculated in Enrichr. (B) barplot showing the enrichment of those genes in Online Mendelian Inheritance in Man database. Bar height indicates statistical significance (−log10 of adjusted p-value) and orange line indicates odds ratio calculated in Enrichr. (C) Barplot showing the enrichment of those genes in VirusMint database. Bar height indicates statistical significance (−log10 of adjusted p-value) and orange line indicates odds ratio calculated in Enrichr. (D) Barplot showing the enrichment of those genes in 2176 housekeeping, 320 tumor suppressor, and 320 oncogenes.

Because PROMINENT uses gene-level DNA methylation data as an input and prioritizes important genes that affect the prediction of diseases, it enables various downstream analyses originally designed for genes, even though we only used methylation data for our analysis. To highlight some of the analyses, we conducted downstream analyses using the top 200 prioritized genes by PROMINENT SHAP values from childhood asthma methylation data (see Supplemental Material) [12]. First, we conducted gene set enrichment analysis (GSEA) using Enrichr [17,18] using the top 200 genes by the SHAP value. In the Panthers database curated in Enrichr, genes were most significantly enriched in the interleukin signaling pathway, confirming our finding that genes in several interleukin pathways are associated with DNA methylation in asthma (Fig. 3A). Angiogenesis is the second most significant pathway. Vascular endothelial growth factor (VEGF), a pivotal angiogenic molecule, is aberrantly expressed in asthma and contributes to bronchial vascular remodeling and chronic inflammation [19]. The third most significant pathway is the Toll-like receptor (TLR)-like pathway, which plays a crucial role in asthma development when it is caused by exposure to environmental triggers such as allergens, microbes, or their byproducts, with TLRs playing a central role [20].

In addition, enrichment analysis using Online Mendelian Inheritance in Man data (OMIM) shows enrichment of various genetic disorders [21]. Among all the diseases, the genes prioritized by PROMINENT were most significantly enriched in asthma, confirming that PROMINENT incorporates highly relevant genes that affect the pathogenesis of a disease to predict disease status (Fig. 3B). This enrichment analysis also highlighted other traits associated with asthma, such as immunodeficiency [22] and obesity [23] (Fig. 3B). Enrichment analysis using the VirusMint database showed enrichment of proteins that interact with various viruses in humans [24]. This analysis is interesting for disease phenotypes that can be triggered or exacerbated by viruses, such as asthma [25] (Fig. 3C). Among the eight significantly enriched types of viruses, four were immunodeficiency viruses, implying that the identified gene-level methylation is not only associated with genotypes, but also with proteins that interact with immunodeficiency.

To investigate the known comorbidity of severe asthma with lung cancer [21], we obtained 320 tumor suppressor genes, 320 oncogenes, and 2176 housekeeping genes from the COSMIC database [26] and tested their enrichment in genes identified by PROMINENT (Fig. 3D) (see Supplemental Material). PROMINENT showed significant enrichment of PROMINENT genes with oncogenes and tumor suppressor genes but not with housekeeping genes. Several oncogenes and tumor suppressor genes have been associated with asthma. For example, chronic lung inflammation, including asthma, inactivates tumor suppressor genes in lung cancer through DNA and protein damage [27]. *YY1* is a key transcription factor that affects asthma [27]. Gene set enrichment analyses enabled by PROMINENT inform the disease-relevant pathways, other relevant diseases, virus-protein interactions, and genes potentially contributing to the disease and cancer comorbidity.

### 2.4. PROMINENT can predict disease phenotypes using the relevant tissue data with a near perfect performance

**Fig. 4.**
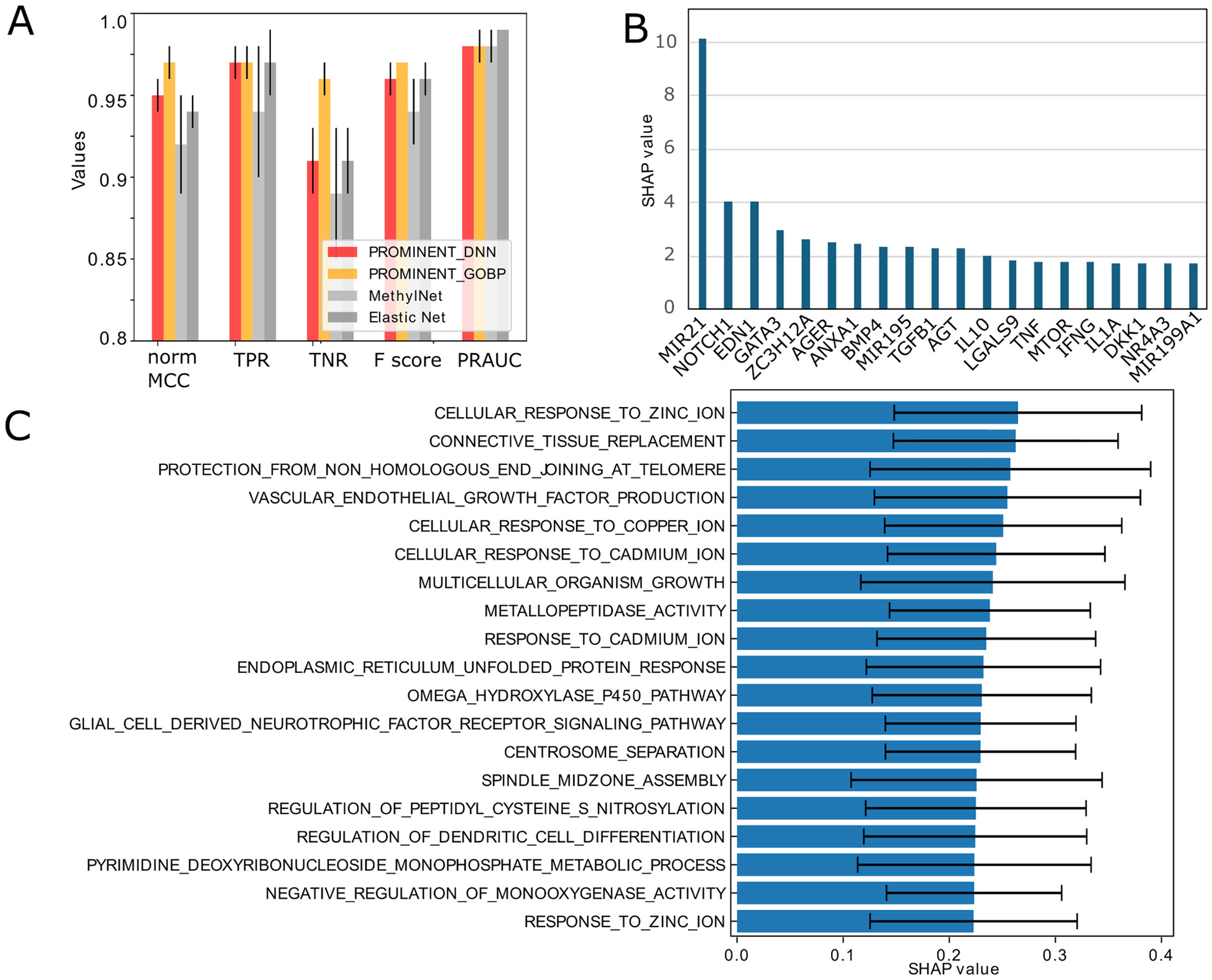
Performance Metrics and Feature Importance in Predicting Idiopathic Pulmonary Fibrosis (IPF) Using PROMINENT and Machine Learning Models. (A) Barplots showing different accuracy metrics to predict IPF vs. normal of PROMINENT_DNN (red), PROMINENT_GOBP (orange), MethylNet (light gray), and Elastic Net (dark gray) on the average of the nested 5-fold CV. Error bars are standard deviation bars based on the nested 5-fold CV experiments. (B) Barplot of SHAP-based importance of gene-level methylation for IFP prediction (the sum of SHAP value across individuals for each gene). (C) Barplot of SHAP-based importance of methylated pathways for the IPF prediction (the sum of SHAP value across individuals for each gene).

To further explore the value of the PROMINENT’s pathway layer, we ran PROMINENT and other methods on the methylation data collected from lung tissues of 345 idiopathic pulmonary fibrosis (IPF) and 202 control samples (GSE175459, see Methods) in 5-fold nested cross-validation experiments. PROMINENT_GOBP showed the best performance for all measures except prAUC (Fig. 4A). In particular, PROMINENT_GOBP outperformed both MethylNet and elastic-net in terms of normMCC and TNR. Due to the high TNR, PROMINENT with GOBP was shown to work best to select genes with strong methylation effects that are associated with the IPF phenotype to guide further experimental validation. Although PROMINENT maintains its advantage in prediction accuracy, it also identifies important genes and pathways aggregated from methylation, which is not available for elastic and MethylNet. To examine the importance of the genes and pathways for IPF, we obtained gene- and pathway-level SHAP values 50 times that of the 5-fold nested cross-validation experiments. We then averaged the SHAP values across 50 experiments to identify the stably prioritized genes and pathways. At the gene level, miRNA-21 was estimated to have the highest SHAP contribution (Fig. 4B). While miRNA-21, a well-known non-coding small RNA, is highly associated with IPF in mouse models [28,29], it is not widely known in human IPF, partly because miRNA-21 is a small and non-coding RNA that functions, especially in the lung. In this study, PROMINENT suggested that miRNA-21 plays a pathological role in human IPF via DNA methylation. Interestingly, *NOTCH1*, which had the third highest SHAP value in childhood asthma, had the second highest SHAP value in IPF because it plays an important role in the pathogenesis of important lung diseases, including pulmonary fibrosis [30]. In addition, the transcription factor *GATA3*, which has the fourth highest SHAP value, upregulates *NRP1* expression and promotes radiation-induced pulmonary fibrosis [31].

Furthermore, PROMINENT underlies the pathological mechanisms of IPF by prioritizing gene pathways. For example, four GO BP pathways within the top 10 pathways based on the SHAP value were related to the cellular response to heavy metal ions (zinc, cadmium, copper, and Fig. 4C, STable 2). A zinc transporter, *ZIP8*, is downregulated in IPF Type 2 alveolar epithelial cells [32]. Additionally, IPF is associated with various metals, including cadmium and copper [32].

Since DNA methylation is a well-known epigenetic mechanism responsible for such environmental exposures [33,34], PROMINENT-based estimation generates reasonable and interesting hypotheses regarding how heavy metal ions can affect IPF by regulating DNA methylation. These findings were possible via pathway-level importance estimation using PROMIENT with a SHAP explainer.

### 2.5. PROMINENT demonstrates a robust and replicable association with phenotypic traits

**Fig. 5.**
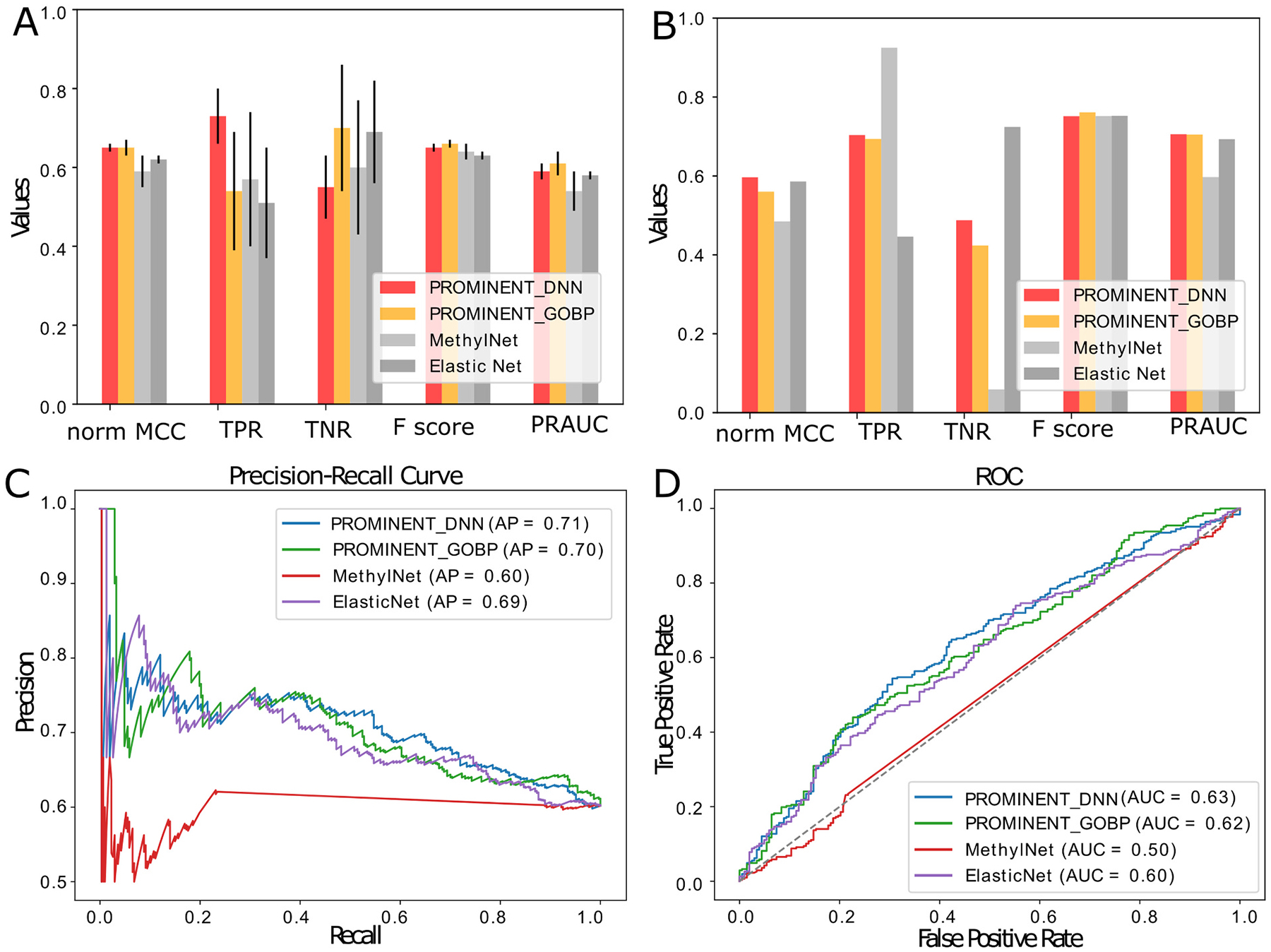
Performance Evaluation of Machine Learning Models in Predicting First-Episode Psychosis (FEP) using PROMINENT_DNN (red), PROMINENT_GOBP (orange), MethylNet (light gray), and Elastic Net (dark gray). (A) Barplots showing different accuracy metrics to predict FEP vs. normal of on the average of the nested 5-fold CV. Error bars are standard deviation bars based on the nested 5-fold CV experiments. (B) Barplots showing different accuracy metrics to predict FEP vs. normal in an independent data set. (C) Precision-recall curve showing prediction performance in the independent data set. (D) ROC curve showing prediction performance in the independent data set.

To measure prediction accuracy not only in held-out test datasets through nested cross-validation, but also in an independent dataset, we obtained the blood DNA methylation data of FEP patients from two independent studies. One study was the European Network of National Schizophrenia Networks Studying Gene-Environment Interactions (EU-GEI) study, which included 413 first-episode psychosis (FEP) patients and 521 controls. Another study was the Institute of Psychiatry, Psychology, and Neuroscience (IoPPN) study, which collected 307 first-episode psychosis (FEP) patients and 203 non-psychiatric controls (see Methods) [35]. We measured prediction accuracy using 5-fold nested cross validation within the EU-GEI cohort data. Both PROMINENT_DNN and PROMINENT_GOBP outperformed MethylNet and elastic net in MCC and PRAUC (Fig. 5A). PROMINENT_GOBP outperformed the other methods in all five measures, except True Positive Rates (TPR). To measure the prediction performance in an independent dataset, we used classification models trained using EU-GEI data to test the IoPPN data (Fig. 5B). The results show that PROMINENT shows a similar range of norm MCC in the test dataset as compared to the 5 fold cross validation data sets.

Interestingly, PROMINENT_GOBP performed better on the test dataset than on the nested-CV dataset in terms of TPR, PRAUC, and F score, possibly because of the larger number of samples in the training set. When we compared the methods, PROMINENT_DNN performed better than the other methods for both MCC and PRAUC. While MethylNET had a very high TPR (0.93), it also had a very low TNR (0.06), showing a very low MCC (−0.03). To draw a precision-recall ROC curve (Fig. 5C) and ROC curve (Fig. 5D) using different threshold values, both variations of PROMINENT performed better than the other methods. Although the performance difference between PROMINENT and elastic net is not substantial, PROMINENT excels in prioritizing both genes and pathways, as demonstrated in previous sections. This capability is not present in elastic net or MethylNet, highlighting PROMINENT’s unique advantage in providing comprehensive insights into biological mechanisms by identifying key genes and pathways involved in disease processes. Altogether, the results show that PROMINENT achieves the best prediction accuracy overall and not only in hold-out datasets but also in an independent test dataset.

### 2.6. PROMINENT enables efficient parameter tuning due to a simpler architecture

To assess the advantages of PROMINENT in terms of computational time, we compared the running times of the four methods in the nested five-fold cross-validation of all the experiments above with the asthma, FEP, and IFP datasets (Table 1). PROMINENT demonstrates a notable advantage over MethylNet, the only other DNN method, across all experiments: PROMIENT_DNN took less than a minute, PROMINENT_GOBP took minutes, and MethylNet took more than 2–3 h to run. For example, to analyze childhood asthma data, PROMINENT_DNN ran 815 times faster than MethylNet and PROMIENT_GOBP ran 46 times faster than MethylNet. PROMINENT_DNN required a comparable time to the Elastic Net model, taking less than a minute for both methods.

To assess the impact of key hyperparameters on model performance, we further evaluated PROMINENT under varying configurations of two major hyperparameters: learning rate and network depth. These parameters are critical in controlling the model’s convergence behavior and its capacity to learn complex representations.

Using the IPF dataset, we trained PROMINENT_GOBP with the following alternative configurations in addition to the baseline 4-layer architecture with a learning rate of 0.01. First, we modified the network depth by (1) adding an extra 32-node dense layer between the merged learning layer and the output layer, resulting in a 5-layer architecture, and (2) removing the merged learning layer to form a 3-layer network (SFig. 3A). Second, we varied the learning rate while keeping the 4-layer architecture fixed, evaluating models trained with learning rates of 0.001 and 0.0001 (SFig. 3B). Across these experiments, only minor differences in performance were observed. The original 4-layer PROMINENT_GOBP model with a learning rate of 0.01 consistently yielded the highest MCC. These results indicate that PROMINENT is robust to moderate variations in network depth and learning rate, maintaining stable performance across different hyperparameter settings.

## 3. Discussion

In our study, we introduced PROMINENT, a novel approach aimed at predicting phenotypic outcomes using DNA methylation data and identifying key genes and pathways associated with phenotypic outcomes. We provided two versions of PROMINENT: PROMINENT without pathway, which is faster but without prioritizing pathways, and PROMIENT with GO pathway, which is bit slower but with prioritizing pathways. We compared the two versions of PROMINENT with a DNN method, MethylNET, and a statistical learning method, Elastic Net, using three publicly available datasets: asthma, IPF, and FEP. Our findings demonstrate PROMINENT’s superior performance over elastic net and MethylNET in these disease predictions, as measured by MCC, across all datasets. In terms of interpretability, PROMINENT excels in prioritizing both individual genes and gene pathways, a capability that lacks in elastic net and MethylNet. The top identified genes and pathways were highly relevant to the respective disease.

The pathway mask assigns gene-level DNA methylation information to their respective biological pathways and further aggregates the gene-level DNA methylation to the pathway level. This pathway-level integration allows the model to consider the collective effect of the genes in each pathway on the disease phenotype. Importantly, this enables the model to interpret the results in the context of biological mechanisms rather than simply treating genes as isolated features. Recently, pathway-informed modeling is gaining momentum as a promising strategy to address the limited biological interpretability of DNA methylation analyses. For example, two independent studies improved the performance of aging and mortality risk predictors compared to individual CpG-based models by aggregating methylation features at the pathway level, such as in inflammation-related pathway [36,37].

While PROMINENT builds upon recent momentum in integrating biological pathways within deep learning frameworks, it advances the field further by incorporating fully connected layers. This design allows to estimate individual gene-level methylation effects to the disease. In addition, it explicitly addresses the inherent incompleteness of current pathway databases by allowing the model to capture important signals from genes not yet annotated in known pathways. The fully connected component serves as a discovery mechanism, enabling PROMINENT to learn from the full breadth of gene-level methylation data, including emerging or under-characterized regulatory elements, thereby enhancing both the flexibility and robustness of phenotype prediction.

To assess the impact of incorporating pathway priors, we compared PROMINENT_GOBP (with pathway constraints) and PROMINENT_DNN (without pathway constraints) across three disease datasets: childhood asthma, idiopathic pulmonary fibrosis (IPF), and first-episode psychosis (FEP). PROMINENT_DNN achieved slightly higher normalized MCC in childhood asthma (Fig. 2A) and in the FEP test dataset (Fig. 5B), suggesting marginally better predictive accuracy in those contexts. This may be because existing pathway databases such as GO and KEGG may omit important or novel pathways, particularly those not yet well-annotated or subject to tissue-specific biases. In contrast, PROMINENT_GOBP demonstrated superior performance in the IPF dataset (Fig. 4A), particularly in terms of MCC and TNR. Importantly, PROMINENT_GOBP enabled the identification of disease-relevant pathways—such as cellular responses to heavy metal ions in IPF (Fig. 4C)—that are not accessible through PROMINENT_DNN.

Given the comparatively higher cost of generating DNA methylation data and its typically smaller sample sizes compared to other data types, such as genotype or electronic health records, selecting an appropriate methodology is crucial for identifying the complexity of the computational model. While simpler models such as the elastic net may excel with smaller sample sizes, more complex models such as MethylNet, which features autoencoder and decoder components, may be preferable for larger datasets. However, because DNA methylation datasets often fall within a medium-sized range, typically comprising hundreds of samples, our simpler DNN-based PROMINENT model was the optimal choice for achieving this balance.

Although the sample sizes required for PROMINENT to achieve optimal prediction accuracy are specific to context (such as tissue type and disease), we conducted experiments by subsampling the IPF dataset with sample sizes ranging from 50 to 500 (see Methods in Supplemental Material). In this disease-relevant tissue (lung), a minimum of 150 samples is required to attain satisfactory prediction accuracy, as measured by the prAUC (see SFig. 4). We anticipate a similar trend if DNA methylation data are collected from disease-specific tissues, such as biopsies of cancer or nasal tissues in asthma.

SMOTE (Synthetic Minority Oversampling Technique) enhances PROMINENT’s performance and usability in three key ways: by addressing explicit class imbalance in the training datasets, by improving prediction even in seemingly balanced datasets with hidden imbalance, and by ensuring the interpretability and biological validity of model training through meaningful synthetic sampling. First, SMOTE directly addresses class imbalance in training data without reducing the total sample size or resorting to naïve duplication of minority cases. This is especially important for our datasets, which display clear imbalances—for example, 345 idiopathic pulmonary fibrosis (IPF) cases and 202 controls, or 307 FEP cases and 203 non-psychiatric controls. By generating synthetic examples in the feature space, SMOTE balances class representation and helps prevent the model from being biased toward the majority class, ultimately improving sensitivity and reducing the misclassification of true positives. Second, SMOTE remains beneficial even when datasets appear balanced in sample count. For example, a dataset may include an equal number of pediatric asthma cases and controls (97 each), yet the distribution of methylation patterns may be more heterogeneous in one class than the other. This hidden imbalance weakens the effective training signal. SMOTE helps PROMINENT overcome this issue by enriching the underrepresented signal in the latent space, leading to improved model calibration and learning efficiency. Third, integrating SMOTE into the pathway-informed PROMINENT architecture supports biological interpretability. Because PROMINENT leverages gene-to-pathway structures and SHAP-based feature attribution, it is essential that any synthetic samples reflect meaningful biological patterns. SMOTE achieves this by interpolating within the minority class, rather than generating arbitrary or duplicated data, ensuring that these synthetic examples contribute constructively to the biologically grounded structure of the network.

Our study underscores the pivotal roles of prioritized genes and pathways by PROMINENT in the pathogenesis of various diseases, suggesting a strong association between DNA methylation and, genes, and also gene pathways. These findings provide compelling evidence that DNA methylation actively contributes to disease pathogenesis. Specifically, our analysis revealed a notable impact of DNA methylation on the immune pathways in childhood asthma. Additionally, we observed associations with pathways responsive to environmental factors, such as heavy metal exposure, further highlighting the relevance of DNA methylation in disease etiology. In previous studies, our expression quantitative trait methylation (eQTM) analysis of childhood asthma revealed a significant enrichment of immune-related genes among those most strongly associated with DNA methylation [38,39]. Similarly, DNA methylation demonstrates superior predictive power for immune genes compared with other genes [40]. Our findings and those of previous studies underscore the importance of studying DNA methylation in immune-related diseases and in those influenced by environmental factors. We suggest using PROMINENT in such studies given its ability to effectively prioritize genes and pathways implicated in disease mechanisms.

PROMINENT demonstrated robust performance across three distinct disease contexts—childhood asthma (blood), idiopathic pulmonary fibrosis (lung), and first-episode psychosis (blood)—outperforming or matching MethylNet and elastic net in key metrics such as MCC, PRAUC, and TNR. Its predictive accuracy held across both cross-validation and an independent validation cohort, supporting generalizability beyond a specific biological context. The consistent identification of biologically relevant genes and pathways across diverse datasets further underscores its broad applicability and reliability in varied biological and epigenetic contexts.

Another interesting finding was PROMIENT’s fast computation time. Despite the expectation of faster computing times for the linear-regression-based Elastic Net model owing to its simpler architecture, the computation time of PROMIENT_DNN is comparable or sometimes even faster than that of the elastic net (Table 1). This efficiency is attributable to the streamlined architecture of PROMINENT_DNN compared with that of MethylNet. Although PROMINENT_GOBP, which incorporates pathway priors, may incur slightly longer computation times than PROMINENT_DNN, the interpretability benefits justify this minor increase for researchers. Because PROMINENT is a simple DNN with only four layers, parameters such as weights and biases are easily optimized with backpropagation and gradient-based optimization (we used the Adam optimizer). The Adam optimizer is a popular algorithm for training deep learning models that combines the benefits of Momentum and RMSProp. It uses two moving averages: one for the gradients (momentum), and the other for the squared gradients (adaptive learning rates). These averages were bias-corrected and used to update the model parameters. Hyperparameters such as layer number and node number are set based on our knowledge, and we believe that further optimization is not required because of its simple structure.

Although PROMINENT generally outperforms the elastic net in terms of prediction accuracy, the difference in some cases, such as with the FEP data, is not very large. However, the true strength of PROMINENT is its ability to provide interpretability. By prioritizing genes and pathways, PROMINENT plays a critical role in biomarker discovery and disease pathogenesis.

While our method, PROMINENT, prioritizes genes and pathways that are important for phenotype prediction, it is essential to highlight its advantages over conventional differential methylation analysis (DMA) followed by pathway enrichment analysis. First, while DMA focuses solely on identifying differentially methylated regions or sites, which is a variable selection problem, PROMINENT addresses both variable section and phenotype prediction simultaneously. Second, whereas most DMA approaches assume a linear association between DNA methylation patterns and phenotypes, PROMINENT can identify nonlinear associations, which are prevalent in many cases [41,42]. Third, although DMA involves multiple sequential steps, testing each CpG site or region individually, PROMINENT streamlines the analysis workflow by prioritizing genes and pathways through SHAP without the need for statistical testing. This reduces the need for multiple sequential steps and avoids the issues related to multiple testing.

The PROMINENT has several limitations. First, the method aggregates CpG-level DNA methylation data at the gene level, enhancing computational efficiency by reducing dimensionality; however, it fails to identify critical CpG sites that may be significant on their own. Second, while employing SHAP for model explanation, it does not perform statistical testing; thus, it does not provide *p*-values that could help establish a clear significance threshold for genes or pathways identified as important. Third, in terms of computational efficiency, PROMINENT is less effective than simpler methods such as elastic net. Finally, the deep neural network architecture of PROMINENT requires hyperparameter tuning, which can be a resource-intensive process. Fourth, the granularity and disease relevance of pathway-level features are inherently dependent on the underlying database. We evaluated PROMINENT using KEGG, REACTOME, GOCC, and GOBP pathways (SFig. 5) and observed notable variability in predictive performance. PROMINENT with GOBP pathways (PROMINENT_GOBP) consistently exhibited superior performance, particularly in normalized MCC and PRAUC. In contrast, PROMINENT with GOCC and with KEGG achieved moderate performance but provided complementary functional and structural insights. These findings highlight the critical role of database selection in shaping model accuracy and interpretability and support the integration of multiple complementary pathway resources. Based on our evaluation of three datasets, we recommend using GOBP database, which consistently outperformed MethylNet and ElasticNet.

While PROMINENT currently predicts only binary outcomes, it has the potential for expansion to accommodate a broader range of outcome types. For example, in multinomial classification scenarios, adjustments of the output layer are required to match the number of classes. Nonetheless, we can continue to utilize Softmax and cross-entropy as the output and loss functions, respectively.

Currently, PROMINENT aggregates CpGs within gene regions. We applied different approaches for summarizing methylation data to improve prediction accuracy. First, we could integrate CpGs significantly associated with genes by incorporating *cis*- and *trans*-expression quantitative trait methylation (eQTM) information [38,39] or expanding the scope to include CpGs within a broader genomic region, such as within 10 Mb of genes, utilizing gene expression prediction methods [40,43]. This enhanced approach aims to incorporate more comprehensive DNA methylation information associated with each gene to better represent gene-level DNA methylation, likely leading to an improved prediction accuracy. Second, instead of focusing solely on gene regions, we would aggregate CpGs within functional regions, such as promoters, gene bodies, 3-′UTRs, 5-UTRs, and enhancers. Because DNA methylation in different functional regions affects gene expression in different ways [38,39], utilizing this detailed information could further refine the model.

In conclusion, we introduced PROMINENT, a gene- and pathway-based neural network model that uses DNA methylation for binary disease prediction. PROMINENT exhibits strong predictive capability, coupled with interpretability and efficient computational time. Therefore, PROMINENT presents a promising avenue for diverse disease-prediction tasks that leverage DNA methylation data.

## 4. Materials and methods

PROMINENT operates through a four-step framework: (1) it pre-processes DNA methylation data by aggregating Beta values at the gene level; (2) these gene-level values are then fed into a deep neural network (DNN) architecture that includes an input layer, a pathway-informed prior layer, fully connected layers, a merged learning layer, and an output layer; (3) the model is optimized using a cross-entropy loss function; and (4) SHAP (SHapley Additive exPlanations) is applied to interpret the model, identifying key genes and pathways predictive of the trait. Each of these steps is described in detail below.

### 4.1. DNA methylation preprocessing and conversion beta values from CpG level to gene level

For all the public DNA methylation datasets used in this study, we performed quality control using the minfi r package to exclude probes that (1) have a detection *p*-value greater than 0.01 in one or more samples; (2) contain single nucleotide polymorphism-introduced artifacts and in cross-reactive regions; (3) have been mapped to multiple locations in the genome; and (4) are mapped to sex chromosomes.

To derive the beta value of each CpG per sample, we used the formula $\beta_{il}=\frac{\text{max}\left(M_{il},0\right)}{\text{max}\left(M_{il},0\right)+\text{max}\left(U_{il},0\right)+\alpha }$, where $M_{il}$ and $U_{il}$ are the methylated and unmethylated intensities of the $l^{\text{th}}$ CpG, respectively, for ith individual [44]. To regularize the beta value when both methylated and unmethylated intensities were low, we added a constant offset a (α=1) to the denominator.

To derive the gene-based methylation beta value, we first annotated CpG sites to gene regions (from promoter to 3′UTR) using the *IlluminaHumanMethylationEPICanno.ilm10b4.hg19* r package for Illumina EPIC methylation data and *IlluminaHumanMethylation450kanno.ilmn12.hg19* r package for Illumina 450 k methylation data. By averaging the methylated and unmethylated intensities of all CpGs annotated to the genes, we calculated the gene-based beta value for gene k and ith individual, as follows:

(1) $$X_{ik}=\frac{\frac{1}{L}\sum_{1}^{L}M_{il}}{\frac{1}{L}\sum_{1}^{L}M_{ij}+\frac{1}{L}\sum_{1}^{L}U_{il}+\alpha }$$

where L CpGs are annotated to gene k. Each CpG, l, has a methylated intensity of Ml and an unmethylated intensity of Ul. By measuring the proportion of the average methylated intensity of L CpGs out of the sum of the average methylated intensity and average unmethylated intensity of L CpGs, we derived the gene-based methylation value. A similar approach has been applied to aggregate methylation beta values to obtain region-level methylation by calculating the mean or median beta values within the region [45]. To regularize the gene-based beta value when both methylated and unmethylated probe intensities were low, we also added a constant offset a (where we set α=1) to the denominator.

### 4.2. Model structure and training of PROMINENT

#### 4.2.1. Input layer

The input layer of PROMINENT takes the matrix X, which consists of $X_{ik}$ such that each row corresponds to each sample, i, whereas each column represents a gene-level methylation beta value for gene k. The MinMaxScaler was used for normalization, ranging from 0 to 1. We set the number of nodes in the input layer as the number of features, that is, the number of genes, K. The input layer is connected to the two parts of the next layer for information extraction.

#### 4.2.2. Pathway prior and (gene-level) fully connected layer

To incorporate the pathway layer into the DNN model, we extended PINNET, which was used to predict Alzheimer’s disease using gene expression data [46].

The second layer of PROMINENT comprises two parts: pathway nodes (p) and fully connected feature integration nodes (f). Pathway nodes (p) indicate the relationships between genes and pathways, whereas fully connected nodes (f) encompass features for all gene-level methylation irrespective of their inclusion in the pathway. The number of nodes in the pathway nodes $(\text{p})\in R^{m}$ equals to the number of pathways utilized.

Between the input layer (genes) and the subsequent layer (pathways), certain connections were masked (assigned a weight of 0). This masking indicates that a specific input node (gene) does not contribute to a certain pathway node, because it is not involved in the biological pathway based on prior knowledge. Consequently, the information at a pathway node is only “flowed” from a specific subset of input nodes (genes) relevant to that pathway.

This design mimics the biological mechanism in which only certain sets of genes interact to drive a specific biological process or function. By assigning weights selectively and masking irrelevant connections, PROMINENT provides a more interpretable and biologically informed representation of the process. This structure ensures that each pathway node aggregates information from its corresponding set of genes, similar to how genes interact with real biological functions.

In this study, we chose the Gene Ontology Biological Process (GOBP), the Gene Ontology Cellular Component (GOCC), REACTOME, the Kyoto Encyclopedia of Genes and Genomes (KEGG) pathway (SFig. 2). The pathway information was downloaded from MSigDB [47,48]. Initially, the GOBP contained 7751 terms, REACTOME contained 1736 gene sets, and GOCC contained 1026 gene sets. To avoid redundant pathway information nodes in the network, we filtered out the pathways if the number of genes that belonged to the pathways was smaller than ten genes.

The pathway nodes were calculated through a fully connected process called binary masking, as shown in Fig. 1B. The pathway nodes that connect the input nodes and biological pathways for individual i are as follows.

(2) $$\left.\left.p_{i}=\text{tanh}\left(\left(W_{p}\circ \text{G}\right)\times X_{i}\right)\right)\circ \text{u}\right)$$

where $p_{i}\in R^{m}$ and $W_{p}\in R^{m\times k}$ is the weight matrices updated by backpropagation. A pathway information matrix $G\in R^{m\times k}$ contains the gene membership information for the pathways, where m is the number of pathways, K is the number of genes, and

(3) $$G_{jk}=\left\{\begin{array}{l} 0\;\text{gene}\;k\notin j\text{th}\;\text{pathway}\\ 1\;\text{gene}\;k\in j\text{th}\;\text{pathway}. \end{array}\right.$$

∘ is element-wise multiplication and therefore $W_{p}\circ \text{G}$ generates a sparse matrix.

$u\in R^{m}$ is a normalization vector designed to mitigate the influence of the number of genes within jth pathway on the values of pathway nodes

(4) $$u_{j}=\frac{1}{\sqrt{\sum_{k=1}^{K}{\text{G}}_{\text{jk}}}}$$

As a complement, we employed 32 fully connected nodes to estimate the effects of individual gene-level methylation, where K input nodes establish direct connections to f via a linear layer, structured as follows:

(5) $${\text{f}}_{i}=\text{tanh}\left(W_{f}X_{i}\right)$$

where $f_{i}\in R^{k}$ and $W_{f}\in R^{k}$ is the weight vectors updated by backpropagation. PROMINENT then concatenates the nodes of $p_{i}$ and $f_{i}$ by column after layer normalization (LayerNorm) in the merged learning layer.

#### 4.2.3. Merged learning layer

PROMINENT fully connects the nodes of the two parts of the second layer to those of the hidden layer. We call the merged learning layer because it integrates information from both parts and, in this way, PROMINENT captures DNA methylation effects at both the pathway and gene levels. Technically, the merged-learning layer is a 64-node dense layer followed by batch normalization, a tanh activation, and a 10 % dropout mask. Batch normalization accelerates convergence, the tanh activation prevents exploding gradient, and the light dropout can help to avoid over-fitting.

#### 4.2.4. Output layer

The merged learning layer is then fully connected to the 2-node output layer. The prediction is output after the softmax function. This indicates the probability of individuals being controlled and cases.

### 4.3. Model optimization

The network was optimized with the Adam algorithm with a learning rate of 0.01 and no weight decay. The loss to minimize is the standard categorical cross-entropy:

(6) $$ℒ(\theta )=-\frac{1}{N}\sum_{i=1}^{N}\sum_{c=1}^{2}y_{ic}\text{log}\left(p_{ic}\left(x_{i};\theta \right)\right)$$

where $p_{ic}$ is the softmax probability that sample i belongs to class c and y∈{0,1} is the binary label.

Training is capped at 200 epochs, but early stopping will be triggered if the validation AUROC doesn’t improve by ≥0.001 for 10 consecutive epochs; the checkpoint with the highest AUROC will be kept as the optimal model.

### 4.4. Model interpretation with SHAP

To explain model f with only a subset S of features, the contribution of the other features is excluded by

(7) $$E\left[f(x)|\text{do}\left(X_{s}=x_{s}\right)\right]$$

In this context, it calculates the expected output of model f, given that the features in subset $X_{S}$ are fixed to specific values $x_{S}$ by the intervention do, and the contributions of all the other features are averaged over. By systematically analyzing all possible subsets of features, SHAP (SHapley Additive exPlanations) values provide a precise and interpretable framework for understanding feature contributions, even in complex models.

As shown in Fig. 1A, we applied the SHAP explainer to the pathway and input layers to prioritize both gene- and pathway-level methylation that contribute to disease prediction. Using nested 5-fold cross-validation (CV), the training set and model were used to fit the explainer, and then the fitted explainer was used to obtain the SHAP values of the features in the test set. After the CV, these SHAP values can be understood as the influence or contribution of the feature nodes to each individual’s predicted outcome. We then calculated feature importance by summing the absolute individual-level SHAP values.

### 4.5. Elastic-Net

For method comparisons, the elastic net [49] was fitted using the gene-level DNA methylation calculated in (1) as covariates, and a phenotype as a binary response, as described in the following equation:

(8) $$\text{logit}(p)=\sum_{k=1}^{K}\hat{\beta }X_{k}$$

where p is the probability of having a disease, $X_{k}$ is the gene-based beta value of gene $k,{\hat{\beta }}_{k}$ is the regression coefficient of gene k, and K is the number of genes.

### 4.6. Hardware specifications

Intel Xeon Platinum 8352Y (Ice Lake) with four nodes, 64 cores/node, 1 TB memory per node, 16 GB memory per core, 10GbE network. For each task, we used one CPU and one GPU (A100 40GB PCIe).

### 4.7. Data selection

To select the dataset for our study, we comprehensively searched the Gene Expression Omnibus (GEO) database (https://www.ncbi.nlm.nih.gov/geo/) for DNA methylation datasets (either 450K or EPIC array) published until August 2023. Our search aimed to identify high-quality, publicly available datasets presenting traits of different etiologies, e.g., psychiatric/neurodegenerative and chronic, for robust training and evaluation of the PROMINENT model. We focused on datasets with a minimum sample size of 100 to ensure that PROMINENT could train the data well, given that deep learning models typically require substantial data to generalize effectively. Additionally, it was crucial that each dataset included both the case and control groups with comparably sized samples to facilitate balanced comparisons and minimize class imbalance, which can otherwise bias model performance and interpretation. We opted not to use TCGA data because of the predominance of tumor samples, which are often paired with a limited number of normal samples, potentially skewing the analysis and limiting the generalizability of the findings. Our extensive search across the entire GEO database was designed to maximize the representativeness and relevance of our study objectives, ensuring a broad and appropriate dataset selection. We did not have any missing data, as we downloaded the data from the public GEO repository, where all missing data were already imputed by the original data submitters. This ensured consistency and completeness across the datasets used in our analyses.

### 4.8. Childhood asthma

PROMINENT was applied to predict the asthma status in the dataset GSE40576, as detailed by Yang [12]. DNA methylation profiles were obtained from peripheral blood mononuclear cells (PBMCs) using the Illumina Human Methylation 450 k BeadChip. This dataset comprised 97 cases, representing children aged 6–12 years with atopy and persistent asthma, and 97 healthy controls. After quality control, 395,899 CpG sites out of a total of 450 K were retained, with 300,460 CpGs situated within the gene regions. The beta values at these CpG sites were aggregated into 20,493 gene-level beta values using Eq. (1).

### 4.9. Idiopathic pulmonary fibrosis (IPF)

PROMINENT was applied to predict Idiopathic Pulmonary Fibrosis (IPF) status and evaluate gene and pathway prioritization in the dataset GSE175459, as detailed by Borie et al. [50]. DNA methylation profiles were obtained from lung tissue samples using the Illumina Human Methylation EPIC system. This dataset was comprised of 345 IPF cases and 202 unaffected controls. After quality control, 661,546 CpG sites out of the total 850 K were retained, with 481,378 CpGs situated within gene regions. The beta values of these CpG sites were aggregated into 24,728 gene-level beta values using Eq. (1).

### 4.10. First episode psychosis (FEP)

To train PROMINENT on one dataset and test it on an independent dataset, we incorporated two independent datasets with first-episode psychosis (FEP). One was from the European Network of National Schizophrenia Networks Studying Gene-Environment Interactions (EU-GEI) cohort (GSE152026), as detailed by Hannon et al. [35]. DNA methylation profiles were obtained from the blood samples of the subjects using the Illumina Human Methylation EPIC Array. This dataset comprised of 413 FEP cases and 521 controls. After quality control, 781,813 CpG sites from 850 K were retained, with 563,429 CpGs situated within gene regions. The beta values of these CpG sites were aggregated into 25,092 gene-level beta values using Eq. (1).

The other dataset was obtained from the Institute of Psychiatry, Psychology, and Neuroscience (IoPPN) cohort (GSE152027), as detailed in the study by Hannon et al. [35], consisting of 307 FEP cases and 203 non-psychiatric controls. DNA methylation of IoPPN samples was obtained using the Illumina Human Methylation 450 k BeadChip. from blood. After quality control, 357,588 CpG sites out of the total 450 K were retained, with 275,754 CpGs situated within the gene regions. The beta values at these CpG sites were aggregated into 18,936 gene-level beta values using Eq. (1). Of the 18,936 genes, 18,929 overlapped with 25,092 genes in the EU-GEI data, and we used these overlapping genes for analysis. We performed a ten-fold CV using the EU-GEI data to select the optimal model with the highest validation AUC, and tested the model using the IoPPN dataset.

### 4.11. Model training and performance evaluation

We used 5-folds cross-validation (CV) to train the model and measure the prediction accuracy. The Synthetic Minority Oversampling Technique (SMOTE) was employed to balance the training sets by over-sampling the minority class without reducing the training sample size [51]. The SMOTE is a popular technique used to address the problem of imbalanced datasets, where one class (e.g., normal samples) has far fewer examples than the other (e.g., disease cases) and runs in the following algorithm. First, select a minority class sample randomly. Second, identify the k-nearest neighbors of the selected point within the minority class using feature similarity. Third, generate synthetic data by randomly choosing one of the neighbors and create a synthetic data point by interpolating between the selected point and its neighbor along the line connecting them. Fourth, repeat this process until the desired number of synthetic minority samples is generated. This approach increases the representation of the minority class by creating diverse synthetic samples rather than duplicating the existing ones. We ran SMOTE with default parameters as follows: sampling_strategy = ‘auto’: resample all classes but the majority class; k_neighbors = 5. The 5 nearest neighbors were used to define the neighborhood of samples to generate the synthetic samples. We used cross-entropy as the loss function and the Adam optimizer to update the model parameters.

Multiple scores were calculated using validation samples to evaluate model prediction. In addition to the area under the precision-recall curve (PRAUC), we computed the Matthews Correlation Coefficient (MCC), true-positive rate (TPR or recall), true-negative rate (TNR or specificity), and F_1_-score. The values were calculated as follows:

(9) $$\text{MCC}=\frac{\text{TP}\cdot \text{TN}-\text{FP}\cdot \text{FN}}{\sqrt{\left(\text{TP}+\text{FP}\right)\left(\text{TP}+\text{FN}\right)\left(\text{TN}+\text{FP}\right)\left(\text{TN}+\text{FN}\right)}}$$

(10) $$\text{TPR}=\frac{\text{TP}}{\text{TP}+\text{FN}}$$

(11) $$\text{TNR}=\frac{\text{TN}}{\text{TN}+\text{FP}}$$

(12) $$F_{1}=\frac{2\text{TP}}{2\text{TP}+\text{FN}+\text{FP}}$$

where TP, TN, FP, and FN represent the true positives, true negatives, false positives, and false negatives, respectively. We selected the threshold that yielded the highest MCC to convert the output probabilities into the predicted classes. F1 depends heavily on TPR because recall (TPR) is a part of its formula.

### 4.12. Hardware specifications

This research was supported in part by the University of Pittsburgh Center for Research Computing and Data, RRID:SCR_022735, through the resources provided. Specifically, this study used the H2P cluster, which is supported by the NSF award number OAC-2117681. For each experiment, we used one core (AMD EPYC 7742) with one GPU (A100 40GB PCIe).

## Supplementary Material

1

2

Supplementary data to this article can be found online at https://doi.org/10.1016/j.artmed.2025.103236.

## Figures and Tables

**Table 1:** Running time (min) of the 4 methods on the Asthma, FEP, and IFP data using nested 5-fold CV.

(min)	Asthma	FEP	IPF
PROMINENT_DNN	0.25	0.55	0.57
PROMINENT_GOBP	4.43	20.05	12.47
MethylNet	203.87	164.48	215.22
Elastic net	0.12	0.797	0.404

## Data Availability

The data underlying this article are available in Gene Expression Omnibus (GEO) at https://www.ncbi.nlm.nih.gov/geo/query/acc.cgi?acc=GSE40576 (Asthma) https://www.ncbi.nlm.nih.gov/geo/query/acc.cgi?acc=GSE175459 (IPF), https://www.ncbi.nlm.nih.gov/geo/query/acc.cgi?acc=GSE152026 and https://www.ncbi.nlm.nih.gov/geo/query/acc.cgi?acc=GSE152027 (FEP).

## Abbreviations

DNAm: DNA methylation
EWAS: Epigenome-wide association studies
MRS: Methylation Risk Scores
MPS: Methylation Profile Scores
DNN: Deep Neural Network
SHAP: SHapley Additive exPlanations
CpG: Cytosine-phosphate-Guanine
GO: Gene Ontology
KEGG: Kyoto Encyclopedia of Genes and Genomes
PROMINENT: Pathway Information on Methylation Analysis using Deep Neural Network
PBMC: Peripheral Blood Mononuclear Cells
IPF: Idiopathic Pulmonary Fibrosis
FEP: First-Episode Psychosis
GOBP: Gene Ontology Biological Process
PRAUC: Precision-Recall Area Under the Curve
norm MCC: Normalized Matthews Correlation Coefficient
TNR: True Negative Rate
eQTM: Expression Quantitative Trait Methylation
GSEA: Gene Set Enrichment Analysis
