# Supplementary material for: Pathway information on methylation analysis using deep neural network (PROMINENT): An interpretable deep learning method with pathway prior for phenotype prediction using gene-level DNA methylation
Source: Artif Intell Med. Author manuscript; Available in PMC 2026 Jun 17. (PMC13273382; doi:10.1016/j.artmed.2025.103236)
Supplement: 1 [file NIHMS2184191-supplement-1.docx]

**Supplemental Materials**

**
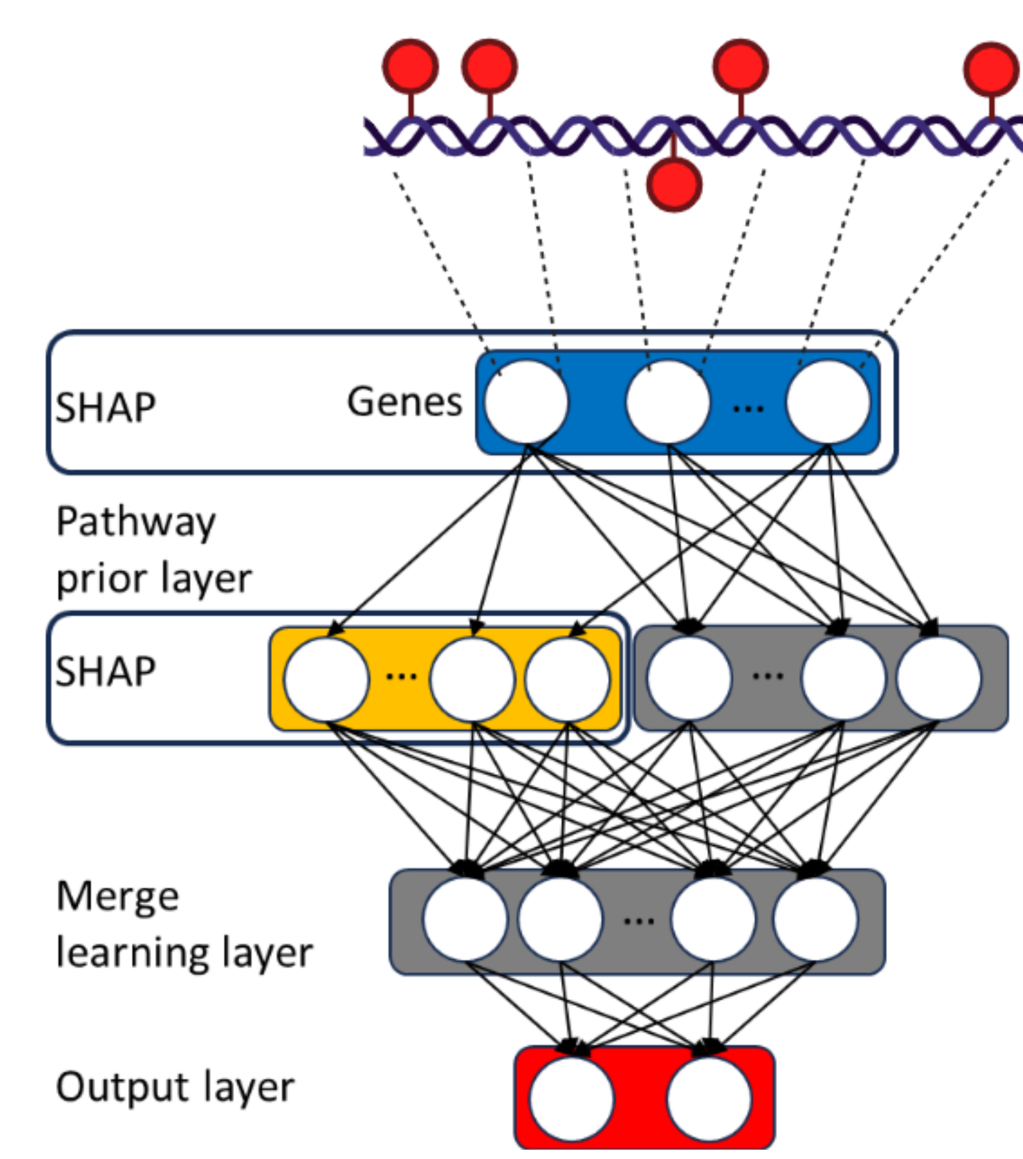
**

**SFig. 1. Prominent overview**


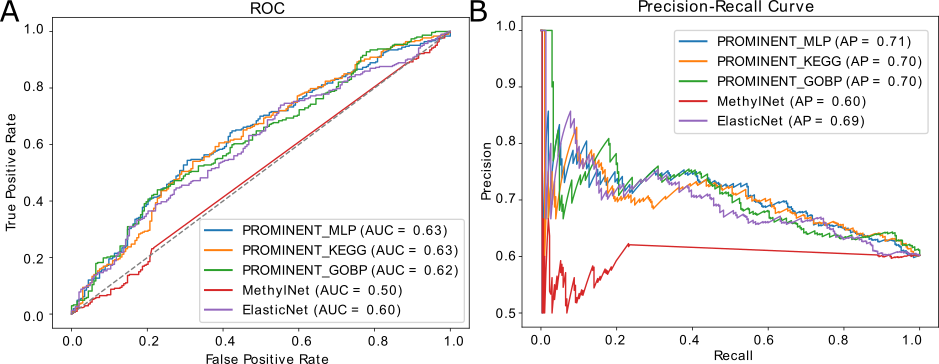


**SFig. 2.** **Performance Evaluation of Machine Learning Models in Predicting First-Episode Psychosis (FEP) including KEGG** (A) ROC curve showing prediction performance in the independent data set. (B) precision-recall curve showing prediction performance in the independent data set.

**
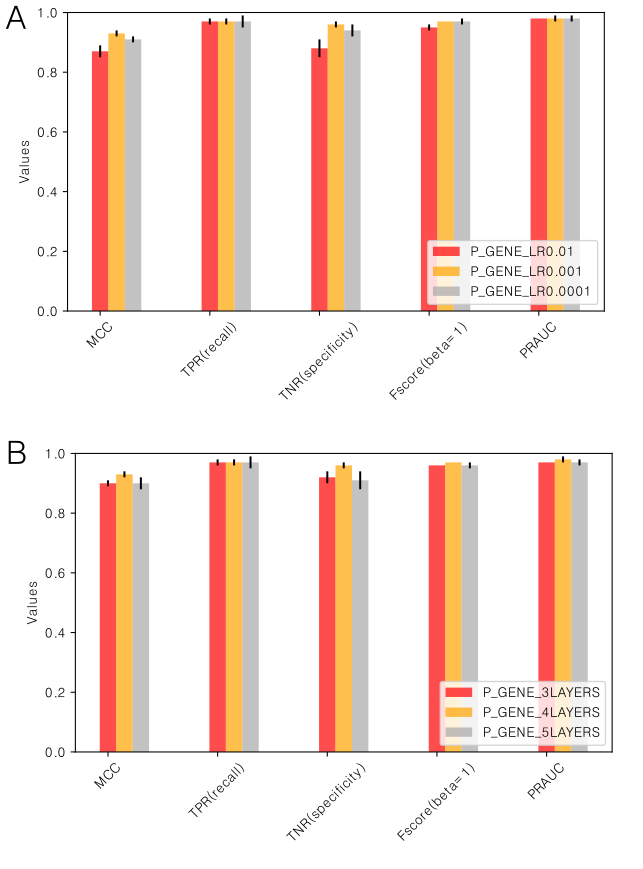
**

**SFig. 3. PROMINENT comparison using different deep learning parameters A. learning rate B. the number of depth**


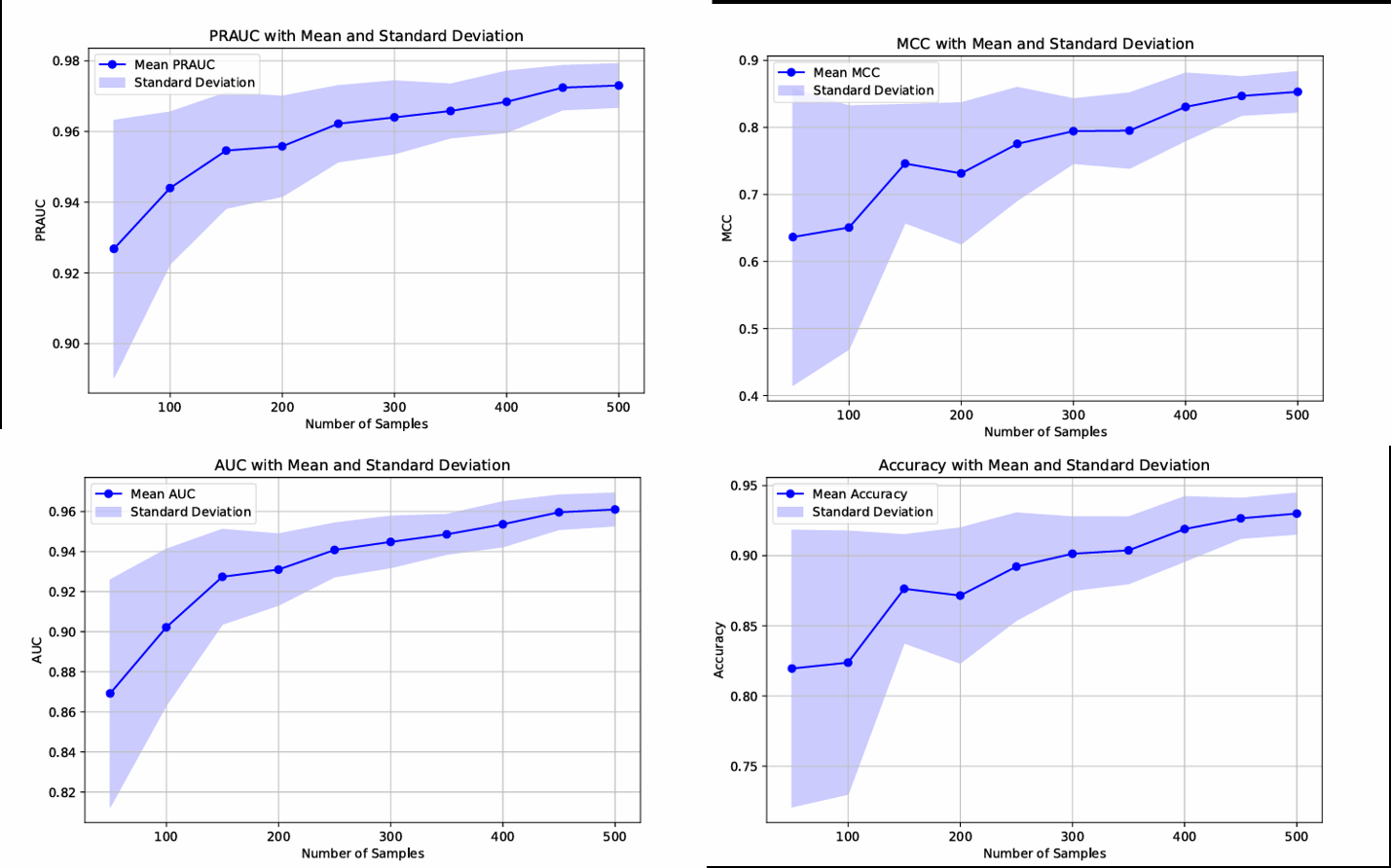


**SFig. 4.** **Performance Evaluation of PROMINENT_GOBP with different sample sizes of IPF data**

**
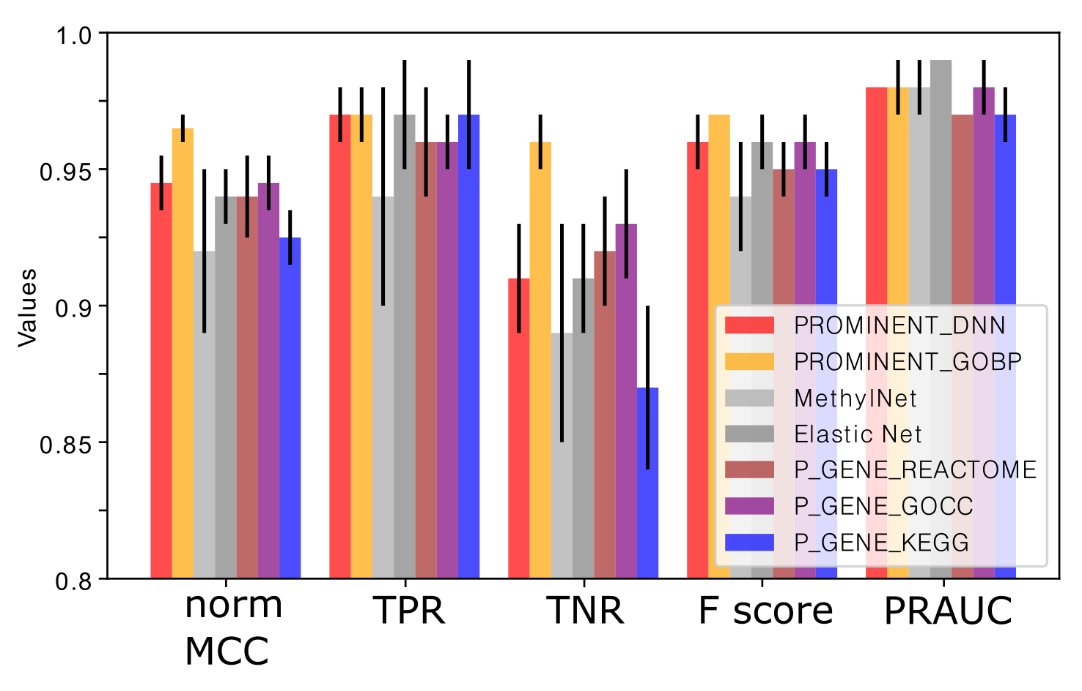
**

**SFig. 5.** **Performance Evaluation of Machine Learning Models in Predicting IPF using PROMINENT_DNN (red), PROMINENT_GOBP (orange), MethylNet (light gray), and Elastic Net (dark gray), PROMINENT_REACTOME (brown), PROMINENT_GOCC(purple), and PROMINENT_KEGG(blue)**. Barplots showing different accuracy metrics to predict IPF vs. normal of on the average of the nested 5-fold CV. Error bars are standard deviation bars based on the nested 5-fold CV experiments.

**Method**

## **Enrichment Analysis**

We conducted multiple gene set analyses using the top genes obtained from PROMINENT, incorporating the GOBP pathway prior (PROMINENT_GOBP) model using childhood asthma data. First, the top 200 gene sets were input into Enrichr [2] to test their enrichment in multiple databases, including Panther, Online Mendelian Inheritance in Man (OMIM), and VirusMint.

To test the enrichment of the 200 genes in tumor-suppressor genes, housekeeping genes, and cancer genes, we utilized the hypergeometric test in scipy.stat in Python. The p-value was calculated using hypergeom.pmf(s, S, q, Q), where s represents the number of successes observed in the sample, S is the total number of successes in the population, Q denotes the number of draws made from the population, and q denotes the size of the sample. We selected tumor suppressor genes (n=320) and oncogenes (K=320) as the sample sets. The population was defined by all the human genes. These data are available at <https://cancer.sanger.ac.uk/census>. We also tested the enrichment of housekeeping genes as controls. We set S=17729.

**STable 1. Machine learning methods utilizing pathway prior**

| **Goal** | **Name(s)** | **Input data** | **Algorithm** |
| --- | --- | --- | --- |
| Prognosis/diagnosis | PASNet [1],  Pathformer [3], IRnet [4] | Transcriptomics data | Hidden layers are connected to gene with pathway hierarchy; weights act as pathway “attention” |
| Subtype stratification | PAVAE/PAAE [5] | Omics panels | Pathway‑regularized latent models using group‑lasso or autoencoders |
| Mechanistic understanding | PathGNN [6],  scapGNN [7] | Transcriptomics data when interactions are important | Graph Neural Networks on pathway graphs |
| *Biomarker selection* | PINNET [8],  Hayakawa et al. [9] | Transcriptomics data | Any DL model, then uses gene‑level importance to gey a ranked list of pathways |

**S Table. 2. Top 30 Genes with the largest absolute SHAP values using asthma data**

| gene | importance | pos | neg |
| --- | --- | --- | --- |
| *IL13* | 1.47448606 | 0.81823587 | 0.65625019 |
| *TLR4* | 0.92342414 | 0.41135523 | 0.51206891 |
| *NOTCH1* | 0.89334975 | 0.33124742 | 0.56210233 |
| *OXTR* | 0.81984133 | 0.40703913 | 0.4128022 |
| *IL4* | 0.80337539 | 0.37649055 | 0.42688484 |
| *LEP* | 0.79420191 | 0.20976107 | 0.58444084 |
| *BRCA1* | 0.78434928 | 0.2277864 | 0.55656289 |
| *EDN1* | 0.76753514 | 0.38336214 | 0.38417301 |
| *CD300A* | 0.72769803 | 0.33027504 | 0.39742299 |
| *IL6* | 0.67531542 | 0.23603739 | 0.43927803 |
| *TNF* | 0.63872615 | 0.29794537 | 0.34078078 |
| *DLGAP5* | 0.60373917 | 0.17808644 | 0.42565272 |
| *WT1* | 0.59582285 | 0.31154573 | 0.28427711 |
| *AGT* | 0.59517141 | 0.19895336 | 0.39621805 |
| *GHRL* | 0.58988362 | 0.34962279 | 0.24026083 |
| *DRD2* | 0.58812309 | 0.22377275 | 0.36435034 |
| *TP53* | 0.58547989 | 0.30002962 | 0.28545028 |
| *AURKB* | 0.5824139 | 0.34190955 | 0.24050435 |
| *WNT3A* | 0.57772375 | 0.28926683 | 0.28845692 |
| *OXT* | 0.57626097 | 0.25878185 | 0.31747912 |
| *CHMP1B* | 0.56021515 | 0.2724745 | 0.28774065 |
| *APOB* | 0.54122729 | 0.33927076 | 0.20195652 |
| *MIR133A1* | 0.54095944 | 0.24058722 | 0.30037222 |
| *HLA-DRB1* | 0.53737093 | 0.25809489 | 0.27927603 |
| *INS* | 0.52940499 | 0.27368151 | 0.25572348 |
| *HTR2A* | 0.51385872 | 0.25118058 | 0.26267813 |
| *SIRT1* | 0.49917741 | 0.29957588 | 0.19960153 |
| *CAT* | 0.48734128 | 0.28189186 | 0.20544942 |
| *NPR2* | 0.47833025 | 0.17003764 | 0.30829261 |
| *APOE* | 0.47777123 | 0.29512832 | 0.18264291 |

## **PROMINENT with Customized Gene Sets**

Researchers can update PROMINENT with updated gene sets (GOBP or KEGG), we provide the following detailed steps:

Download Gene Sets: Users can download the gene sets of interest from MSigDB in .gmt format, which is widely used for pathway and gene set analysis.

Prepare Input Data: Once the gene sets are obtained, users can prepare their data using the following PROMINENT command:

PROMINENT-data_prepare --input_csv <filename> --input_gmt <filename> --output <filename>

Here:

<filename> for --input_csv should point to the user’s input data file containing features (e.g., gene-level methylation values).

<filename> for --input_gmt should refer to the downloaded gene sets in .gmt format.

<filename> for --output specifies the desired name for the processed output file.

Gene Nomenclature Consistency: It is crucial that the gene features in the input CSV file and the gene identifiers in the .gmt file use the same nomenclature, such as HUGO gene names. This ensures that the mapping between input features and gene sets is accurate.

Further Guidance: Additional details and examples for using PROMINENT can be found in the official GitHub repository: <https://github.com/cloudmacchiato/PROMINENT/tree/main/example>

**Model Training and Performance Evaluation**

To evaluate the effect of sample size on the prediction of PROMINENT, we did a down sampling experiment with PROMINENT_GOBP and IPF data. Specifically, to avoid biases, a random stratified sampling was used to get several subsets of the samples with the same case/control ratio and we did 50 runs of training and testing for each subset. These models are used for the evaluation of the effect of the sample size on PROMINENT (**Fig. S2**).

1. Hao, J., et al., *PASNet: pathway-associated sparse deep neural network for prognosis prediction from high-throughput data.* BMC Bioinformatics, 2018. **19**(1): p. 510.

2. Xie, Z., et al., *Gene Set Knowledge Discovery with Enrichr.* Current Protocols, 2021. **1**(3): p. e90.

3. Liu, X., et al., *Pathformer: a biological pathway informed transformer for disease diagnosis and prognosis using multi-omics data.* Bioinformatics, 2024. **40**(5).

4. Jiang, Y., et al., *IRnet: Immunotherapy response prediction using pathway knowledge-informed graph neural network.* Journal of Advanced Research, 2025. **72**: p. 319-331.

5. Avelar, P.H.d.C., et al. *Pathway Activity Autoencoders for Enhanced Omics Analysis and Clinical Interpretability*. in *2024 IEEE International Conference on Bioinformatics and Biomedicine (BIBM)*. 2024.

6. Liang, B., et al., *Risk stratification and pathway analysis based on graph neural network and interpretable algorithm.* BMC Bioinformatics, 2022. **23**(1): p. 394.

7. Han, X., et al., *scapGNN: A graph neural network–based framework for active pathway and gene module inference from single-cell multi-omics data.* PLOS Biology, 2023. **21**(11): p. e3002369.

8. Kim, Y. and H. Lee, *PINNet: a deep neural network with pathway prior knowledge for Alzheimer's disease.* Front Aging Neurosci, 2023. **15**: p. 1126156.

9. Hayakawa, J., et al., *Pathway importance by graph convolutional network and Shapley additive explanations in gene expression phenotype of diffuse large B-cell lymphoma.* PLOS ONE, 2022. **17**(6): p. e0269570.
